# Robust estimation of SARS-CoV-2 epidemic in US counties

**DOI:** 10.1038/s41598-021-90195-6

**Published:** 2021-06-04

**Authors:** Hanmo Li, Mengyang Gu

**Affiliations:** Department of Statistics and Applied Probability, University of California, Santa Barbara, CA 93106 USA

**Keywords:** Infectious diseases, Statistics

## Abstract

The COVID-19 outbreak is asynchronous in US counties. Mitigating the COVID-19 transmission requires not only the state and federal level order of protective measures such as social distancing and testing, but also public awareness of time-dependent risk and reactions at county and community levels. We propose a robust approach to estimate the heterogeneous progression of SARS-CoV-2 at all US counties having no less than 2 COVID-19 associated deaths, and we use the daily probability of contracting (PoC) SARS-CoV-2 for a susceptible individual to quantify the risk of SARS-CoV-2 transmission in a community. We found that shortening by $5\%$ of the infectious period of SARS-CoV-2 can reduce around $39\%$ (or 78 K, $95\%$ CI: [66 K , 89 K ]) of the COVID-19 associated deaths in the US as of 20 September 2020. Our findings also indicate that reducing infection and deaths by a shortened infectious period is more pronounced for areas with the effective reproduction number close to 1, suggesting that testing should be used along with other mitigation measures, such as social distancing and facial mask-wearing, to reduce the transmission rate. Our deliverable includes a dynamic county-level map for local officials to determine optimal policy responses and for the public to better understand the risk of contracting SARS-CoV-2 on each day.

## Introduction

The outbreak of new coronavirus 2019 (COVID-19) has caused nearly 200,000 deaths in the US, and among those, there are 2277 counties with no less than 2 associated deaths as of 20 September 2020^1^. The ongoing COVID-19 pandemic has led to unprecedented non-pharmaceutical interventions (NPIs), including travel restrictions, lockdowns, social distancing, facial masks wearing, and quarantine to reduce the spread of SARS-CoV-2 in the US. The COVID-19 outbreak is prolonged and asynchronous across regions. Thus it is critical to estimate the dynamics of COVID-19 epidemic to determine appropriate protective measures before the availability of effective vaccines.

A non-negligible proportion of SARS-CoV-2 infectious individuals is asymptomatic or have mild symptoms^2^. We term the individuals the *active infectious individuals* who can transmit the disease to others but may not be diagnosed yet. Identifying the number of active infectious individuals is crucial to monitor the transmission in a community. Another important time-dependent quantity is the expected number of secondary cases resulted from each active infectious individual, or *effective reproduction number*. In this article, we estimate these two time-dependent quantities for all US counties with no less than 2 COVID-19 associated deaths as of 20 September 2020; the population of some counties that falls within this category is even less than ten thousand. Furthermore, based on these two time-dependent quantities, a more interpretable measure, called the daily *probability of contracting* (PoC) SARS-CoV-2 for an individual at the county-level was used to quantify the risk. This static risk factor with fixed transmission rates was studied before^3^. Here we studied the dynamic transmission rate parameter, which is estimated by the number of deaths, test positive rates and the number of confirmed cases in a community. The risk factor can be extended to measure the risk of an event with different sizes^4^. The fine-grain estimation of disease progression characteristics allows the public to understand the risk of contracting COVID-19 on a daily basis.

Predictive mathematical models are useful for analyzing an epidemic to guide policy responses^5^. The epidemiology compartmental models such as SIR, SEIR, SIRD, and their extensions^6–10^, stochastic agent based models^11,12^, branching processes^13^, and network analysis^14^ have advanced our understanding of transmission rates and incubation period of SARS-CoV-2, which are connected to the traffic flow and mobility during the COVID-19 outbreaks at different regions^15,16^. The disease progression characteristics, such as the transmission rate, are often estimated based on the daily death toll^6,9,11,12^. However, it is challenging to estimate the progression of the epidemic in US counties with small population, because the number of daily observed confirmed cases and COVID-19-related deaths is small.

Meanwhile, using observed laboratory-confirmed COVID-19 cases (henceforth, observed confirmed cases) might significantly underestimate the population that have been infected with the SARS-CoV-2. It was found in Ref.^17^ that around $9.3\%$ of the US individuals (or roughly 30 million) may have contracted the COVID by July 2020 based on serology tests, whereas less than 4.8 million COVID-19 positive cases have been confirmed in the US before August 2020^1^. Thus, it is important to estimate the number of individuals who contracted COVID-19 but had not tested positive. The focus herein is on integrating COVID-19-related death toll and test data to obtain a robust estimation of the disease progression characteristics of COVID-19 at county and community levels.

One critical quantity to evaluate an infectious disease outbreak is the time-dependent transmission rate, based on which one can compute the basic reproduction number and the effective reproduction number of the disease. Various approaches were proposed to estimate this parameter. The transmission rate was modeled as a decreasing function of the time in Ref.^6^, a function of NPIs in Ref.^11^ and a geometric Brownian motion in^18^. Unlike the outbreak in China or other countries in north-east Asia, transmission rates of the COVID-19 progression in the US does not monotonically decrease due to the prolonged duration of the outbreak, and it is challenging to determine a suitable parametric form of this parameter in terms of time. In Ref.^9^, the transmission rate parameter was related to the initial values of infectious cases, resolving cases, and up to two derivatives of the daily death toll. This method provides a flexible way to estimate the time-dependent transmission rate from the death toll and its derivatives, yet unstable for counties with moderate or small population sizes, as numerical estimation of the daily death toll and its derivatives is often unstable.

**Figure 1:**
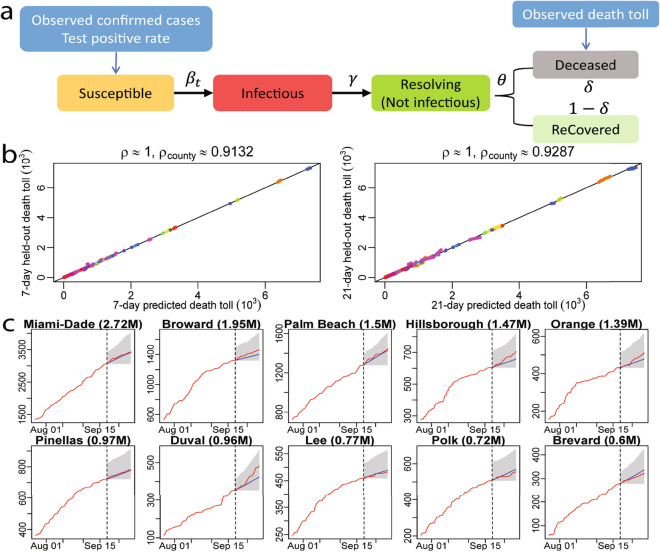
(**a**) The SIRDC model and the data used for analysis. (**b**) 7-day death toll forecast and 21-day death toll forecast against the held-out truth in 2277 US counties with no less than 2 deaths as of 20 September 2020. Each dot is a cumulative death toll for one county at one held-out day. Counties from the same state are graphed using the same color. The Pearson correlation coefficient ($\rho$) of the nation and the weighted average of Pearson correlation coefficient for counties ($\rho _{county}$) are recorded. (**c**) 21-day death toll forecasts in 10 counties with largest population in Florida, where the red line represents the observed death toll and blue line means the forecast. The forecast starts from 21 September 2020, marked by the vertical black dash line. The grey shadow area is the 95$\%$ confidence interval of the forecast. Numbers in the parentheses right after the county name are population in million. The Figs. 2 and 3 show 21-day death toll forecast for all counties in Florida and California.

In this work, we propose a robust approach of integrating test data and death toll to estimate COVID-19 transmission characteristics by a Susceptible, Infectious, Resolving (but not infectious), Deceased, and reCovered (SIRDC) model initially studied in^9^. We illustrate that the transition between different stages of disease progression in the SIRDC model in part a of Fig. 1. First, a part of the population is infected by active infectious individuals each day, depending on the transmission rate parameter ($\beta _t$). After $\gamma ^{-1}$ days, an active infectious individual is expected to be no longer infectious, denoted by the resolving compartment, meaning that this individual will not transmit COVID-19 to others as a result of hospitalization or self-quarantine. We term the average length of an active infectious individual the *infectious period*. A resolving case is expected to be resolved (either recovered or deceased) after $\theta ^{-1}$ days. The proportion of deaths from the number of resolved cases is controlled by the fatality rate parameter $\delta$.

**Figure 2:**
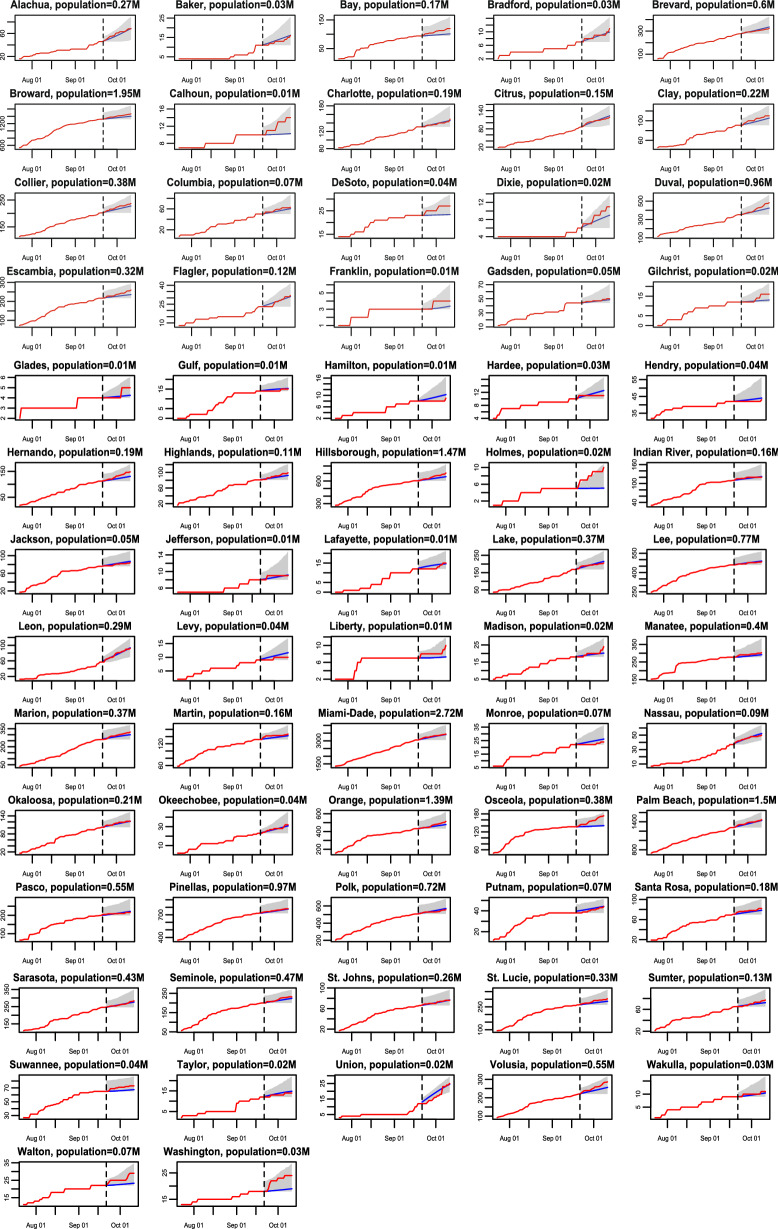
The 21-day forecast in 67 Florida counties with death toll no less than 2 as of 20 September 2020. The training period is from 21 March 2020 to 20 September 2020, whereas the forecast starts from 21 September 2020. The red curves are the cumulative observed death toll from 21 September 2020 to 11 October 2020 and the blue line indicates the forecast for the same period. The shaded area represents the $95\%$ predictive intervals of the forecast for each analyzed county in Florida.

**Figure 3:**
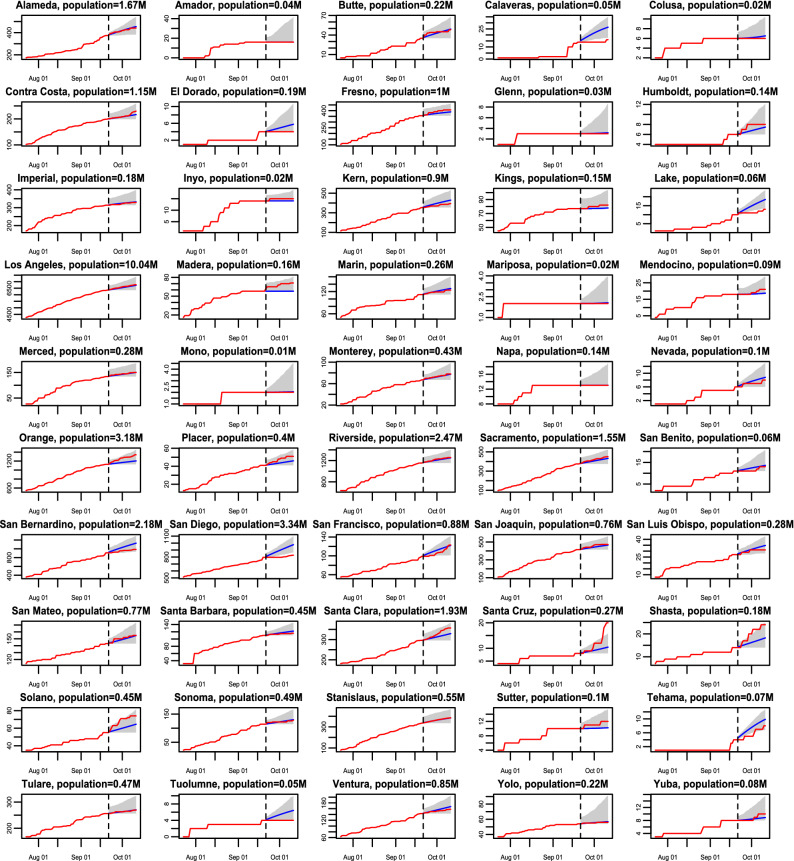
The 21-day forecast in 50 California counties with death toll no less than 2 as of 20 September 2020. The training period is from 21 March 2020 to 20 September 2020, whereas the forecast starts from 21 September 2020. The red curves are the cumulative observed death toll from 21 September 2020 to 11 October 2020 and the blue line indicates the forecast for the same period. The shaded area represents the $95\%$ predictive intervals of the forecast for each analyzed county in California.

**Figure 4:**
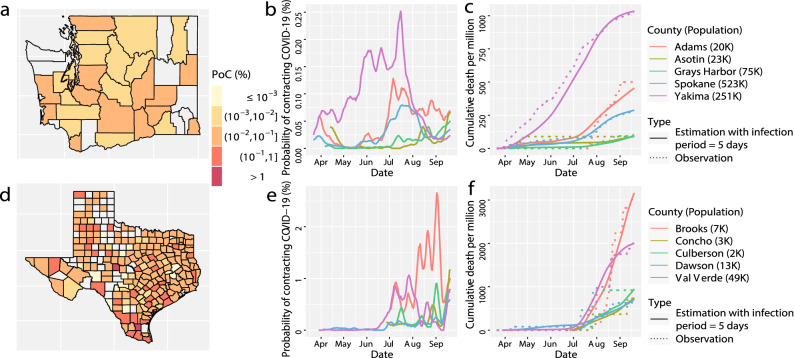
(**a**) The estimated probability of contracting SARS-CoV-2 in Washington state on 20 September 2020. (**b**) the probability of contracting SARS-CoV-2 from 5 counties in Washington state with the largest PoC SARS-CoV-2 values on 20 September 2020 . (**c**) the observed (dots) and fitted (solid line) cumulative death toll in the 5 counties in figure (**b**) from the same time period. (**d**–**f**) The results in Texas that have the same interpretation as (**a**–**c**). Part (**e**) and (**f**) have different scales than part (**b**) and (**c**), respectively.

**Figure 5:**
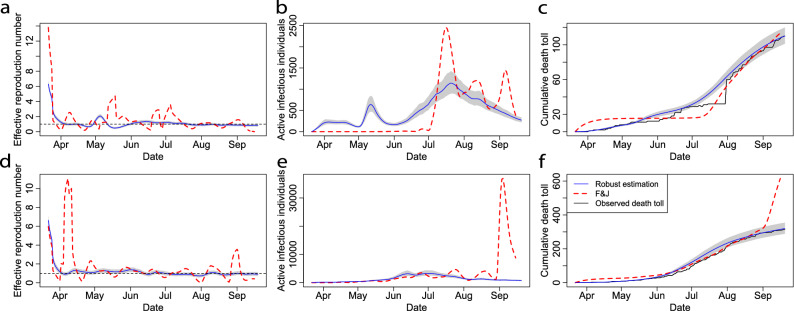
(**a**–**c**) Comparisons between the estimation COVID-19 progression characteristics for Santa Barbara, CA as of 20 September 2020 by our algorithm 1 (blue solid curves) and the method $F \& J$^9^ (red dash curves). The shaded area represents $95\%$ confidence intervals. The black solid curve in part c is the observed cumulative death toll in Santa Barbara. (**d**–**f**) Results for Imperial, CA as of 20 September 2020, which have the same interpretation as (**a**–**c**). The transmission rate estimated from the method $F \& J$ is truncated to be within [0,10].

**Figure 6:**
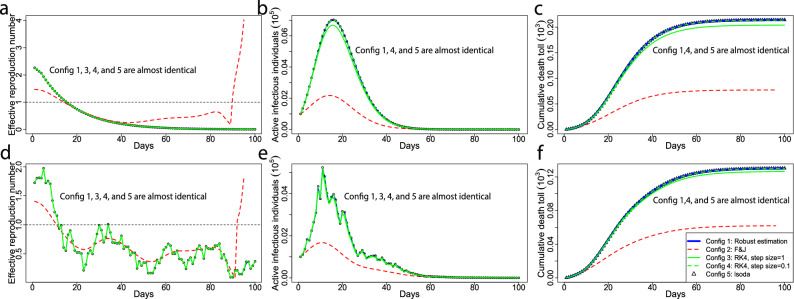
(**a**–**c**) Simulated comparison with noise-free observations. The black circles are the solution of the ODEs of the SIRDC model via the default numerical solver Isoda in the function ode in deSolve R package. The green solid and dash curves are the numerical solutions from Runge–Kutta method with the 4th order integration and step size being 1 and 0.1, respectively. The Blue solid curves are the robust estimation from algorithm 1 and red dash curves are the estimation in^9^. In the simulation with noise-free observations, we let time duration be $T=100$ days, the population size $N=10^{7}$, the initial values of 5 compartments chosen as $(S(1), I(1), R(1), D(1), C(1))= (N-2000, 1000, 1000, 0, 0)$ and the transmission rate $\beta (t) = \text{ exp }\left( -0.7 (\frac{9}{T-1} (t-1) + 1) \right)$, for $1\le t \le T$. (**d**–**f**) results of the simulation with noisy observations, which have the same interpretation as (**a**–**c**). In this simulation, we set the transmission rate $\beta (t) = \text{ exp }\left( -0.7 (\frac{9}{T-1} (t-1) + 1) \right) + \epsilon$, for $1\le t \le T$ and $\epsilon \sim N(0, 0.04)$, and the other parameters are held the same as in the noise-free simulation. The transmission rates estimated from the method $F \& J$ are truncated to be within [0,10]. The solution from our robust estimation approach, the Isoda and the Runge–Kutta method with the 4th order and step size being 0.1 overlap for both scenarios.

Our approach has three innovations. First, we solve the compartmental models using a midpoint rule with a step size of 1 day, as the confirmed cases and death toll are updated daily in most US counties, and this is discussed in the method section. Second, we combine test positive rates, confirmed cases and death toll to estimate the daily transmission rate parameter. Our estimate of transmission rates and reproduction numbers is robust and accurate to reproduce the number of the death toll and other compartments for counties with medium to small population sizes (Figs. 4 and 5). The simulated studies also suggest that our approach is more robust than the solution in Ref.^9^ (Fig. 6), as our solution does not require estimating derivatives of the daily death toll. Only two parameters, the initial values of the number of active infectious individuals and the number of resolving cases, need to be estimated numerically for each county. Then we can solve the time-dependent transmission rates and all other compartments subsequently. Since only two parameters are estimated for each county, our estimation rarely depends on initial values we choose for the optimization. Finally, we use a Gaussian process to model the residual between the observed death toll and that from the SIRDC model, leading to more accurate predictions and proper uncertainty quantification. A summary of the main findings, limitations, and policy implication are given in Table 1.

**Table 1:** Policy summary.

Background	The transmission of SARS-CoV-2 is heterogeneous and asynchronous in US counties. It is thus important to assess the risk before lifting or replacing any mitigation measure in the community. We have developed a novel approach to integrate test data and death toll to estimate the probability of contracting COVID-19, as well as the time-dependent transmission rate and number of active infectious individuals at the county level in the US
Main findings and limitations	National level order of protective measures reduces the transmission rate and active number of infectious individuals for most US counties in April, whereas the risk of contracting SARS-CoV-2 rebounded between late June and early July, as the protective measures were relaxed. We found that when the infectious period of SARS-CoV-2 is shortened by $5\%$ and $10\%$, the number of deaths can be reduced from 199 K to 120 K ($95\%$ CI [109 K, 132 K ]) and 80 K ($95\%$ CI [72 K, 89 K]) as of 20 September 2020, respectively, when other protective measures were kept the same. The reduction of the infectious period can be achieved by extra testing in addition to ongoing protective measures. Our model relies on the existing knowledge of the COVID-19 and model assumptions. Other information, such as demographic profiles, mobility, and serology test data, can be used to calibrate the model parameters and assumptions at the community level.
Policy implications	Our model indicates that extra testings, along with the current NPIs, can significantly reduce the number of deaths associated with COVID-19. The estimated probability of contracting COVID-19 can be used as an interpretable risk factor to guide community policy responses.

## Results

**Figure 7:**
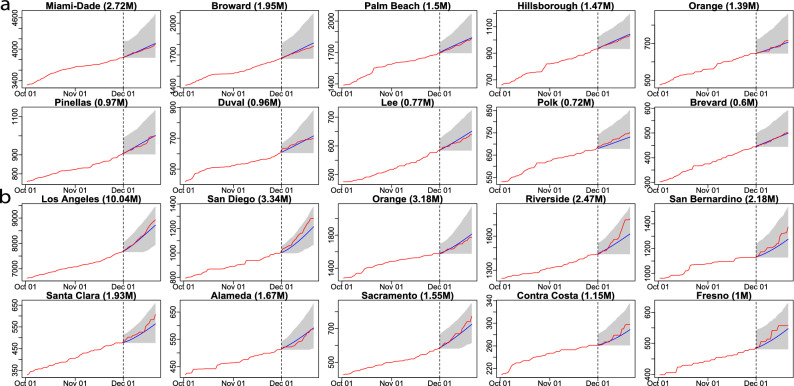
(**a**) The 21-day forecast in 10 counties with the largest population in Florida. The training period is from 21 March 2020 to 30 November 2020, whereas the forecast starts from 1 December 2020. The red curves are the cumulative observed death toll and the blue line indicates the forecast from 1 December 2020 to 21 December 2020. The shaded area represents the $95\%$ predictive intervals of the forecast for each analyzed county in Florida. The numbers in the parentheses are the populations in million for each county. (**b**) the 21-day forecast in 10 counties with the largest population in California. The interpretations are the same as (**a**).

**Figure 8:**
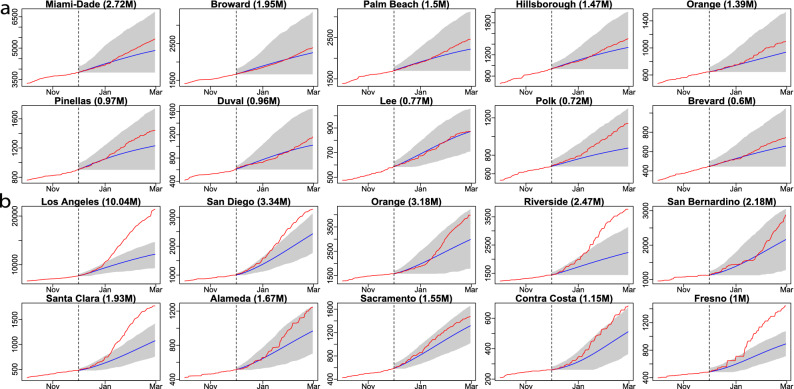
(**a**) The 90-day forecast in 10 counties with the largest population in Florida. The training period is from 21 March 2020 to 30 November 2020, whereas the forecast starts from 1 December 2020. The red curves are the cumulative observed death toll and the blue line indicates the forecast from 1 December 2020 to 28 February 2021. The shaded area represents the $95\%$ predictive intervals of the forecast for each analyzed county in Florida. The numbers in the parentheses are the populations in million for each county. (**b**) the 90-day forecast in 10 counties with the largest population in California. The interpretations are the same as (**a**).

We first verify our model performance by forecasting at the county level. The 7-day and 21-day death projections for 2277 US counties using data by 20 September 2020, for instance, are close to the held-out test death toll in these counties, shown in part b and part c of Fig. 1. The Pearson correlation coefficient ($\rho$) is larger than 0.999 7-day and 21-day forecast. We also calculate the weighted average of Pearson correlation coefficient for counties ($\rho _{county}$), which treats each county as a different population and population size is used to computed the weighted average of Pearson correlation coefficient for counties. The 21-day forecast of each considered county in Florida and California using observations by 20 September 2020 is provided in Figs. 2 and 3 , respectively. The death toll forecast based on our model is accurate for most US counties, and around $95\%$ of the held-out test data is covered by nominal $95\%$ predictive interval (Supplementary Table S1 in supplementary information), indicating that the uncertainty assessment is accurate. To further test the predictive performance of our model, we use data by 1 December, 2020 to make 21-day and 90-day predictions of deaths in the 10 largest counties in Florida and California. The forecast results are shown in Figs. 7 and 8 , respectively. While this is a challenging scenario, as confirmed cases and deaths increase dramatically across the US during the winter, we found that our 21-day predictions are reasonably accurate for all 20 counties. Thus, our models can be used reliably for the short-term projection of COVID-19 related deaths at the county level during different periods of the epidemic. Furthermore, a 90-day accurate forecast of US counties before the winter may be an almost impossible task, and indeed we underestimate death counts for a few counties due to a rapid increase in death counts during the winter. On the other hand, our model that fuses test data and death toll correctly projects the rapid increase in death counts for most counties during the winter, even if death counts do not increase dramatically during the training period.

Based on the robust estimation of transmission rates, we derived the county-level estimation of daily PoC SARS-CoV-2. We classify the daily PoC SARS-CoV-2 in a community into five levels listed in Table 2. On 20 September 2020, out of 2277 US counties, only 60 counties were at the controllable level and 311 counties were at the moderate level, whereas 1906 counties were at the either alarming, strongly alarming, or hazardous level. The daily PoC SARS-CoV-2 measures the average probability to contract SARS-CoV-2 for a susceptible individual in a community, and the risk varies from individuals to individuals. Nonetheless, the PoC SARS-CoV-2 is an interpretable measure for public understanding of the average risk of contracting SARS-CoV-2 in a community on a given day.

**Table 2:** Interpretation of the daily PoC SARS-CoV-2 in a community.

Daily PoC SARS-CoV-2	$<0.001\%$	$0.001\%$ to $0.01\%$	$0.01\%$ to $0.1\%$	$0.1\%$ to $1\%$	$>1\%$
Risk	Controllable	Moderate	Alarming	Strongly alarming	Hazardous

**Figure 9:**
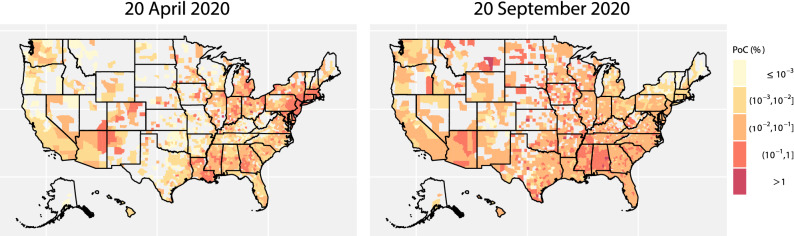
(**a**) The estimated probability of contracting SARS-CoV-2 at 1856 counties on 2020-04-20, and (**b**) at 2277 counties on 20 September 2020. The probability of contracting SARS-CoV-2 is truncated at $10^{-6}$, whereas only 78 counties on 20 April and 45 counties on 20 September are below this level, respectively.

We graph the estimated PoC SARS-CoV-2 of an individual at US counties on 20 April 2020 and 20 September 2020 in Fig. 9. On 20 April 2020, the PoC SARS-CoV-2 is large in northeastern regions and some southern states such as Arizona, New Mexico, and New Orleans. On 20 September 2020, the PoC SARS-CoV-2 is large in many inland states, for instance, Montana, North Dakota, Mississippi, and Alabama. Although the PoC SARS-CoV-2 on 20 September in northeastern regions is substantially lower than that on 20 April, the PoC SARS-CoV-2 for an individual is large in most other states on 20 September, suggesting that the relaxation of protective measures can lead to more population contracting COVID-19, and consequently more deaths at a rate no slower than that in late April.

Officials can use the daily PoC SARS-CoV-2 to determine whether the mitigation policies can be lifted or replaced by other measures for different regions. The probability of contracting COVID-19 in many counties in Texas on 20 September 2020, for example, is larger than those in Washington [(part (a) and (d) in Fig. 4], indicating that Texas should undertake more protective measures to reduce the risk. The nationwide lockdown order and social distancing in spring effectively reduced the PoC SARS-CoV-2 in 4 out of 5 counties in Washington, while the PoC SARS-CoV-2 of all counties increases in late June and early July, as some of the nonpharmaceutical interventions (NPIs) were lifted (part b in Fig. 4). Part (c) shows that the model fits the death toll. With only two parameters estimated numerically for each county, the fit is reasonably good for these counties at a wide range of dates. In comparison, though the outbreak of 5 counties in Texas started in early summer, the PoC SARS-CoV-2 in these Texas counties is much higher than that in Washington counties on 20 September [part (e) in Fig. 4]. Our model also fits the death toll of the counties in Texas relatively well [part (f) in Fig. 4]. The county-level estimation and forecast are updated regularly on the COVID-19 US Dashboard:https://covid19-study.pstat.ucsb.edu/.

**Figure 10:**
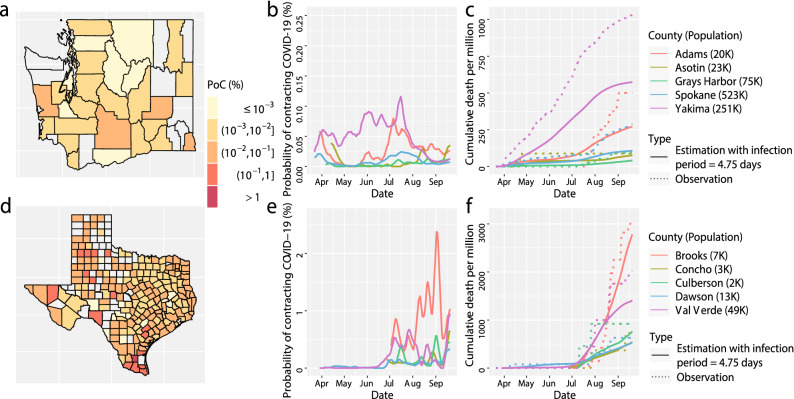
(**a**–**f**) The simulated results of COVID-19 progression in Washington (the first row) and in Texas (the second row) that have the same interpretation as (**a**–**f**) in Fig. 4 with the infection period changed from 5 days, to 4.75 days, whereas other parameters are held the same.

**Figure 11:**
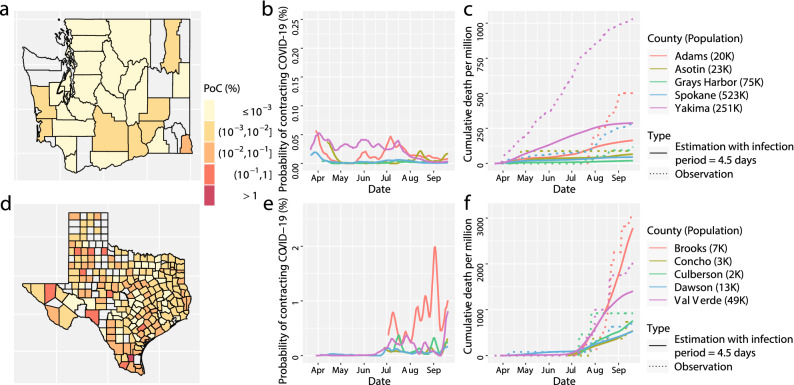
(**a**–**f**) The simulated results of COVID-19 progression characteristics in Washington (the first row) and in Texas (the second row) that have the same interpretation as (**a**–**f**) in Fig. 4 with the infection period changed from 5 to 4.5 days, whereas other parameters are held the same.

The effectiveness of protective measures were studied to reduce the transmission rate^7,8,11,12,14,19^, whereas the efficacy of these measures depends on the reactions from the public, which is likely to vary from region to region. Another simultaneous effort to mitigate the spread of the COVID-19 outbreak is through testing and contact tracing, which reduces the infectious period, and consequently, the number of active infectious individuals. For Washington and Texas, we simulate the model output with infectious period reduced by $5\%$ (or equivalently 4.75 days in total), while the transmission rate ($\beta _t$ in SIRDC model) is held the same. We found that the PoC SARS-CoV-2 is reduced by 5 times for 12 counties out of 28 considered counties in Washington and 6 counties out of 209 considered counties in Texas, as shown in the Fig. 10. Furthermore, when we reduce the infectious period by $10\%$ (or equivalently 4.5 days in total), while the transmission rate ($\beta _t$ in SIRDC model) is held the same, the PoC SARS-CoV-2 is reduced by 5 times for 26 out of 28 counties in Washington and 146 out of 209 counties in Texas, shown in Fig. 11.

**Figure 12:**
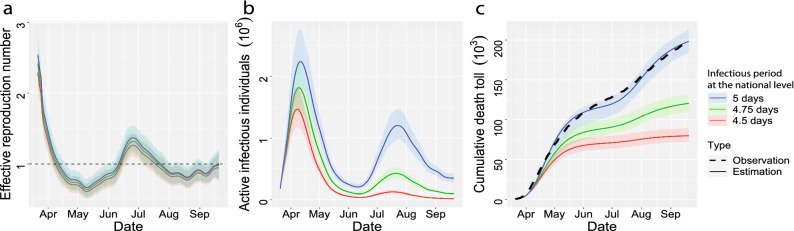
(**a**,**b**) The estimate reproduction number and overall number of active infective individuals in the US, including 50 states and Washington D.C., from 21 March 2020 to 20 September 2020 with infectious period assumed to be 5 days (blue), 4.75 days (green) and 4.5 days (red). (**c**) The estimate overall death toll in the US. The time period and interpretation of (**c**) are aligned with a and (**b**), except that the black dots in (**c**) stand for the observed death toll in the US.

**Figure 13:**
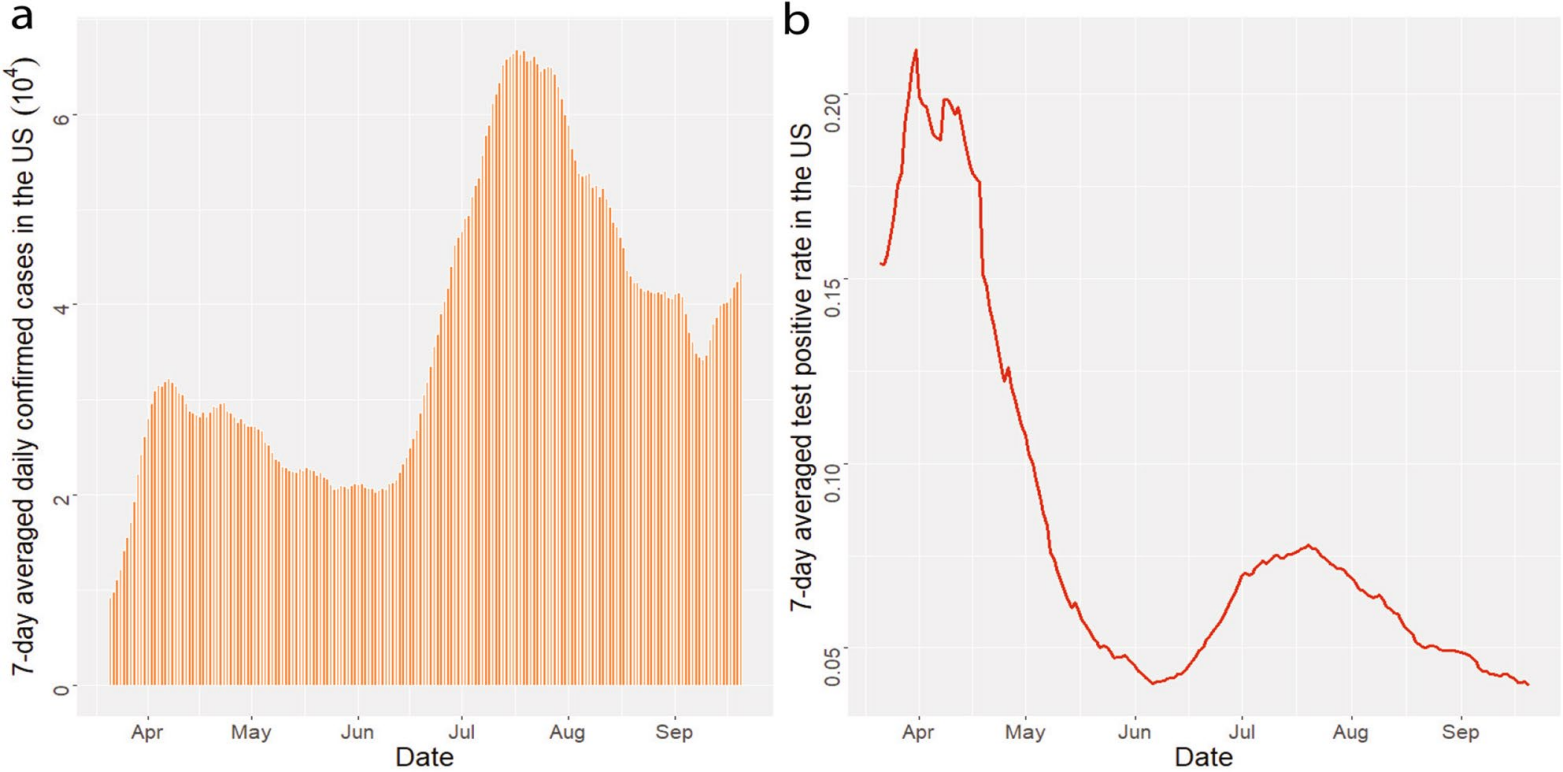
(**a**) The 7-day averaged daily confirmed cases in the US from 21 March 2020 to 20 September 2020. (**b**) the 7-day averaged test positive rate in the US from 21 March 2020 to 20 September 2020.

We graph the estimated effective reproduction number, the number of active infectious individuals, and the cumulative death toll in the US, along with the simulated values when the average infectious period is reduced from 5 to 4.75 days and 4.5 days in Fig. 12. First, we found that mitigation measures in March effectively reduce the effective reproduction number to below 1, whereas the value rebounded in summer after some of these measures were relaxed in different regions. Consequently, the US has experienced two waves of the outbreak in terms of the number of active infectious individuals [part (b) in Fig. 12]. The high test positive rate at the beginning of the epidemic (Fig. 13) indicates that a substantial number of active infectious individuals were not diagnosed in April due to the lack of diagnostic tests. According to our estimates, the peak of the first wave in April is larger than that of the second wave in July in terms of the number of active infectious individuals, whereas the peak of the daily observed confirmed cases in April is smaller than that of the second wave in July (Fig. 13).

Second, the simulated results suggest that shortening infectious period of SARS-CoV-2 by $5\%$ and $10\%$ can reduce the total deaths from 199 K to 120 K ($95\%$ CI [109 K, 132 K]) and 80 K ($95\%$ CI [72 K, 89 K]), respectively, as of 20 September 2020, when other protective measures were held as the same (part c in Fig. 12). Note that since we held the transmission rate parameter ($\beta _t$) to be the same (a scenario where the public adheres to the protective measure same as the reality), the effective reproduction number barely changes (part a in Fig. 12). However, the slightly shortened infectious periods of SARS-CoV-2 can reduce the death toll substantially (part c in Fig. 12), as the number of active infectious individuals decreases (part b in Fig. 12).

We found that a shortened infectious period substantially reduces the number of active infectious individuals and fatalities in the second wave. However, the changes are smaller in the first wave, since the effective reproduction number in the second wave is smaller than that in the first wave (Fig. 12). The county level estimation also validates this point (Figs. 10 and 11). This finding indicates that the efforts of shortening the infectious period of SARS-CoV-2 should not replace the other protective measures, such as social distancing and facial mask-wearing to reduce the transmission rate.

Diagnostic tests can be used to shorten the length of the infectious period of an active infectious individual. Drastically reducing the infectious period may not be possible without contact tracing, which is challenging when there is a large number of active infective cases. Reducing the infectious period by around $5\%$, in comparison, may be achieved by periodically diagnostic tests every 20 days for each susceptible individual. More frequent testing or contact tracing may be needed to achieve this goal, as the infection is most likely to happen between days 2 and 6 after exposure due to the high viral load of SARS-CoV-2^20^. Another efficient way is to test susceptible individuals with a high risk of contracting or spreading SARS-CoV-2, such as individuals with more daily contacts or have contacts with vulnerable populations, e.g., workers from senior living facilities. Our estimation of the PoC SARS-CoV-2 can be used as a response to develop regression models using covariates including demographic information and mobility to elicit personalized risk of contracting SARS-CoV-2 for susceptible individuals.

Finally, efforts on reducing the length of the infectious period should not replace other protective measures for reducing transmission rates of SARS-CoV-2, as the number of active infectious individuals and death toll can be effectively reduced only if the effective reproduction number is not substantially larger than 1.

## Discussion

Our study has several limitations. First, our findings are based on the available knowledge and model assumptions, as with all other studies. One critical parameter is the death rate, assumed to be $0.66\%$ on average^21^, whereas this parameter can vary across regions due to the demographic profile of the population and available medical resources. The studies of the prevalence of SARS-CoV-2 antibodies based on serology tests^17^ can be used to determine the size of the population who have contracted SARS-CoV-2, and thus provides estimates on the death rate, as the death toll is observed. Besides, we assume the infected population can develop immunity since recovery for a few months, which is commonly used in other models. The exact duration of immunity post-infection, however, remains unverified scientifically. Third, we assume that the number of susceptible individuals and, consequently, the number of individuals who have contracted SARS-CoV-2 can be written as a function of the number of observed confirmed cases and test positive rates, calibrated based on the death toll. More information such as the proportion of population adhere to the mitigation measures, mobility, and demographic profile can be used to improve the estimation of susceptible individuals in a region.

Our results can be used to mitigate the ongoing pandemic of SARS-COV-2 and other infectious disease outbreaks in the future. The estimated daily PoC SARS-CoV-2 at the county level, for example, is an interpretable measure to understand the risk of contracting COVID-19 on a daily basis and a surveillance marker to determine appropriate policy responses. Besides, Our method can be extended when an effective vaccine becomes available^10^. Finally, further studies of this measure relative to different mobility, demographic information, and social-economic status can provide more precise guidance for local officials to protect vulnerable populations from contracting SARS-CoV-2, when an effective vaccine is not available.

## Methods

We introduce our methods in this section. The main symbols used in this section and their definitions are provided in Table 3.

**Table 3:** Main symbols and definitions in the “Methods” section.

Symbol	Definition
*S*(*t*)	Number of susceptible cases on day t
*I*(*t*)	Number of infectious cases which can transmit COVID-19 on day t
*R*(*t*)	Number of resolved cases which get infected but cannot transmit COVID-19 on day t
*D*(*t*)	Number of deceased cases on day t
*C*(*t*)	Number of recovered cases on day t
*N*	Number of population in a given area
$\beta (t)$	Transmission rate on day t
${\gamma }^{-1}$	Average number of days an individual can transmit COVID-19
${\theta }^{-1}$	Average number of days for a case to get resolved
$\delta$	Proportion of deceased cases, a.k.a. fatality rate
$R_0(t)$	Basic reproduction number on day t
$R_{eff}(t)$	Effective reproduction number on day t
*P*(*t*)	Average probability of contracting (PoC) SARS-CoV-2 on day t
*p*(*t*)	State-level test positive rate on day t
$c^o(t)$	Cumulative number of observed confirmed cases on day t
$\Delta c^o(t)$	Daily number of observed confirmed cases on day t
$c^u(t)$	Cumulative number of unobserved confirmed cases on day t
$\alpha$	Power parameter for estimating the number of susceptible cases
$\omega$	Weight parameter for estimating the number of susceptible cases
*z*	Zero-mean Gaussian process

### SIRDC compartmental models

The SIRDC model for the *j*th county in the *i*th state in the US is described below:

1 $$\begin{aligned} \begin{aligned} {\dot{S}}_{i,j}(t)&= \frac{-\beta _{i,j}(t) S_{i,j}(t) I_{i,j}(t)}{N_{i,j}}, \\ {\dot{I}}_{i,j}(t)&=\frac{\beta _{i,j}(t) S_{i,j}(t) I_{i,j}(t)}{ N_{i,j}}-{\gamma I_{i,j}(t)}, \\ {\dot{R}}_{i,j}(t)&= {\gamma I_{i,j}(t)}-{\theta R_{i,j}(t)}, \\ {\dot{D}}_{i,j}(t)&={\delta \theta R_{i,j}(t)},\\ {\dot{C}}_{i,j}(t)&={(1-\delta ) \theta R_{i,j}(t)}, \end{aligned} \end{aligned}$$

where $S_{i,j}(t)$, $I_{i,j}(t)$, $R_{i,j}(t)$, $D_{i,j}(t)$ and $C_{i,j}(t)$ denote the number of individuals at these 5 compartmental groups on day *t*, respectively, and $N_{i,j}$ denotes the number of individuals in county *j* from state *i* for $i=1,2,\ldots ,k$, $j=1,2,\ldots ,n_{i}$ with $n_i$ being the number of counties of the *i*th state considered in the analysis and $t=1,2,\ldots ,T_{i,j}$. The time-dependent transmission rate parameter is denoted by $\beta _{i,j}(t)$ and the inverse of average number of days an infectious individual can transmit the COVID-19 is denoted by $\gamma$. The inverse of the average number of dates for a case to get resolved (i.e. deceased or recovered) is denoted by $\theta$ and the proportion of deceased cases (i.e. death rate) is denoted by $\delta$. The parameters $(\gamma , \theta , \delta )$ were invariant over time and held fixed in this study. Following^19^, we assume the infectious period to be 5 days on average, and a case is expected to resolve after 10 days. The average death rate is assumed to be $0.66\%$^21^. Additional verification of these assumptions and sensitivity analysis of these parameters are provided in the supplementary information.

To determine the characteristics of the SARS-CoV-2 epidemic in US counties, we define the time-dependent *effective reproduction number*, i.e. the average number of secondary cases per primary cases as ${\fancyscript{R}}_{eff}^{i,j}(t)={\fancyscript{R}}_{0}^{i,j}(t)S_{i,j}(t)/N_{i,j}$, where the ${\fancyscript{R}}_{0}^{i,j}(t)=\beta _{i,j}(t)/\gamma$ denotes the *basic reproduction number* on day *t*. When ${\fancyscript{R}}_{eff}^{i,j}(t)<1$, it means that the number of the active infectious individuals will decrease (and vice versa, if ${\fancyscript{R}}_{eff}^{i,j}(t)>1$). The effective reproduction number was often used to quantify whether or not the disease is under control^22^. However, the effective reproduction number does not directly quantify risk of contracting SARS-COV-2 for a susceptible individual, as the number of active infectious individuals in a region was not taken into consideration. We compute the average probability of contracting (PoC) SARS-CoV-2, denoted as $P_{i,j}(t)={{\fancyscript{R}}^{i,j}_{eff}(t)I_{i,j}(t)}\gamma /({S_{i,j}(t)})={\mathcal \beta _{i,j}(t)I_{i,j}(t)}/{N_{i,j}}$, which quantifies the risk of a susceptible individual in county *j* from state *i* to catch SARS-CoV-2 on day *t*. Here the risk is on an average sense among all susceptible individuals in a region.

The most critical parameter of the SIRDC model is the transmission rate parameter, $\beta _{i,j}(t)$, as a function of time, based on which we obtain the reproduction number on day *t*. To estimate the time-dependent transmission rates for communities with small population sizes, we derive a more robust estimation of the transmission rate of each county based on the death toll and testing data, discussed below.

### Closed-form expressions of the time-dependent transmission rates

Since the observations such as death toll and confirmed cases are generally updated daily, we solve the ordinary differential equations (ODEs) in the SIRDC model (Eq. 1) approximately by the midpoint rule of the integral with a step size of 1 day. For day $t \in {\mathbb {N}}^{+}$, the approximation is described below:

2 $$\begin{aligned} \frac{S_{i,j}(t+1)}{S_{i,j}(t)} \doteq&~\exp \left\{ -\frac{\beta _{i,j}(t+0.5) }{2N_{i,j}} \left( I_{i,j}(t) + I_{i,j}(t+1)\right) \right\} , \end{aligned}$$

3 $$\begin{aligned} \frac{I_{i,j}(t+1)}{I_{i,j}(t)} \doteq&~\exp \left\{ \frac{\beta _{i,j}(t+0.5)}{2N_{i,j}} (S_{i,j}(t) + S_{i,j}(t+1)) - \gamma \right\} , \end{aligned}$$

4 $$\begin{aligned} R_{i,j}(t+1) - R_{i,j}(t) \doteq&~\gamma \frac{I_{i,j}(t) + I_{i,j}(t+1)}{2} - \theta \frac{R_{i,j}(t) + R_{i,j}(t+1)}{2}, \end{aligned}$$

5 $$\begin{aligned} D_{i,j}(t+1) - D_{i,j}(t) \doteq&~\delta \theta \frac{R_{i,j}(t) + R_{i,j}(t+1)}{2}, \end{aligned}$$

6 $$\begin{aligned} C_{i,j}(t+1) - C_{i,j}(t) \doteq&~(1-\delta ) \theta \frac{R_{i,j}(t) + R_{i,j}(t+1)}{2}. \end{aligned}$$

Further by assuming the transmission rate parameter $\beta _{i,j}(t)$ is day-to-day invariant (i.e. a step function with step size 1), based on Eqs. (2) and (3), we obtain $\beta _{i,j}(t+0.5)$ from $t = 1$ to $T_{i,j}-1$, iteratively, based on the sequence of susceptible individuals $\{S_{i,j}(t)\}_{t=1}^{T_{i,j}}$ and the initial number of active infectious individuals $I_{i,j}(1)$ described in algorithm 1.

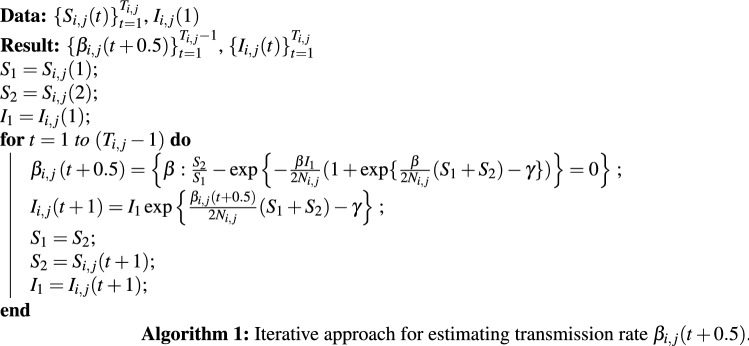



After we get the number of active infective individuals ($I_{i,j}(t)$) on each day, sequences of the resolving, deceased and recovered compartments can be solved subsequently following the same manner using Eqs. (4)–(6), after specifying their initial values. Expressing the time-dependent transmission rate by the number of susceptive and infective cases is the key to integrating death toll and testing data for estimation.

In Figs. 5 and 6, we demonstrate that in order to solve the ODEs in the SIRDC model, our approach is more accurate and robust than the method $F \& J$ in Ref.^9^ under both simulated and real scenarios. Other more accurate methods (such as the Runge–Kutta method) can also solve the ODEs of SIRDC model, but the time-dependent transmission rates can not easily be expressed as a function of the death toll and the number of active infectious individuals as the way they are in our solution.

### Estimation of the number of susceptible individuals

Note that we have $S_{i,j}(t) + c_{i,j}^o(t) + c_{i,j}^u(t) = N_{i,j}$ for any *t*, where $c_{i,j}^o(t)$ and $c_{i,j}^u(t)$ are the number of cumulative observed confirmed cases and unobserved confirmed cases, respectively. Estimating the number of susceptible individuals is equivalent to estimating the number of unobserved confirmed cases $c_{i,j}^u(t)$, because the number of observed confirmed cases $c_{i,j}^o(t)$ and the population $N_{i,j}$ are known. Here we combine them with the positive test rates to estimate $c_{i,j}^u(t)$, as large positive test rates typically indicate a large number of unobserved confirmed cases. We assume that the total number of confirmed cases is equal to the observed confirmed cases, adjusted by the state-level test positive rate $p_{i}(t)$, a power parameter $\alpha _i$ and a weight parameter $\omega _{i,j}$, leading to the following formula of the susceptible population:

7 $$\begin{aligned} S_{i,j}(t) = N_{i,j} - c_{i,j}^o(t) - c_{i,j}^u(t) = N_{i,j} - \frac{1}{\omega _{i,j}} \left\{ {\mathbb {1}}_{ \{t \ge 2\}} \sum _{s=2}^{t} (p_i (s))^{\alpha _i} \Delta c_{i,j}^o(s) + {(p_i (1))^{\alpha _i}} c_{i,j}^o(1) \right\} , \end{aligned}$$

where $\Delta c_{i,j}^o(t)$ is the observed daily confirmed cases on day *t*, for $t=1,2,\ldots ,T_{i,j}$, $i=1,2,\ldots ,k$ and $j=1,2,\ldots ,n_{i}$. Since the positive test rates are only available at the state level, the power parameter $\alpha _i \in [0,2]$ is estimated by the state-level observations. According to Eq. (7), the time-invariant weight $\omega _{i,j}$ can be expressed below:

8 $$\begin{aligned} \omega _{i,j} = \frac{{(p_i (1))^{\alpha _i}} c_{i,j}^o(1)}{I_{i,j}(1)+R_{i,j}(1)+D_{i,j}(1)+C_{i,j}(1)}, \end{aligned}$$

where $I_{i,j}(1)$, $R_{i,j}(1)$, $D_{i,j}(1)$ and $C_{i,j}(1)$ are the number of active infectious, resolving, deceased and recovered cases on day 1, respectively.

### Estimation of initial values of infectious and resolving cases

We define day 1 of a county as the more recent date between 21 March 2020 and the date that the county has 5 observed confirmed cases for the first time. Since all counties were at an early stage of the epidemic on the starting day, we let the initial value of the death toll $D_{i,j}(1)$ be the observed death toll on the day 1, and the initial value of the recovered cases be 0. This assumption is not likely going to strongly influence our analysis, as the number of recovered cases is only a negligible proportion of the susceptible individual on the starting day if not zero. The only parameters to estimate are the number of infectious individuals $I_{i,j}(1)$ and the number of resolving cases $R_{i,j}(1)$ on the day 1 for county *j* from state *i*, after the power parameter $\alpha _i$ is estimated using the state-level observations to minimize the same loss function below:

9 $$\begin{aligned} \begin{aligned} ({\hat{I}}_{i,j}(1), {\hat{R}}_{i,j}(1))&= {{\,\mathrm{arg\,min}\,}}\sum _{t=1}^{T_{i,j}} \left( \frac{{D_{i,j}(t) - \hat{D}_{i,j}(t \mid I_{i,j}(1), R_{i,j}(1)) }}{T_{i,j}-t+1}\right) ^2, \, s.t. \\ 0&\le I_{i,j}(1)+R_{i,j}(1) \le U_{i,j},\, I_{i,j}(1) \ge 0, \text{ and } R_{i,j}(1) \ge 0, \end{aligned} \end{aligned}$$

where the upper bound $U_{i,j}$ is chosen to guarantee the estimated number of the susceptible cases $S_{i,j}(t)$ to be larger than 0:

$$\begin{aligned} U_{i,j}&= N_{i,j} \frac{{(p_i (1))^{\alpha _i}} c_{i,j}^o(1)}{{\mathbb{1}}_{\{T_{i,j} \ge 2\}} \sum _{s=2}^{T_{i,j}} (p_{i}(s))^{\alpha _{i}} \Delta c_{i,j}^o(s) +{(p_i (1))^{\alpha _i}} c_{i,j}^o(1)} - (D_{i,j}(1) + C_{i,j}(1)), \end{aligned}$$

for $$t=1,2,\dots , T_{i,j}$$.

After the initial values of infectious and resolving cases are estimated, we obtain the estimation of the susceptible cases from Eq. (7), and the infectious cases and transmission rates on each date for each county from Algorithm 1. The resolving cases, deaths, and recovered cases can be derived subsequently from Eqs. (4)–(6), respectively. The estimated basic and effective reproduction rates can be derived by the fitted time-dependent transmission rate, and the estimated probability of contracting SARS-CoV-2 for an individual can be computed based on transmission rate and number of infectious individuals for each county on each day.

### Forecast and uncertainty assessment

Our method can also be used as a tool for forecasting compartments (e.g., death toll), reproduction numbers, and the probability of contracting SARS-CoV-2 at each county for a short period. We extrapolate the transmission rate based on Gaussian processes implemented in RobustGaSP R package^23^ with robust parameter estimation^24,25^. Based on the extrapolated transmission rates, the compartments can be solved iteratively based on Eqs. (2)–(6).

We also found that the forecast will generally be improved by modeling residuals between observed deaths and modeled deaths by a zero-mean Gaussian process (GP). One advantage of a GP model is the internal assessment of the uncertainty of the forecast from the predictive distribution, which is of crucial importance. The aggregated model that combines the SIRDC model and the GP model for county *j* from state *i* in the US is described as follows.

10 $$\begin{aligned} D_{i, j}(t) = F_{i,j}(t) + z_{i,j}(t) + \varepsilon _{i,j,t}, \end{aligned}$$

where $D_{i,j}(t)$ and $F_{i,j}(t)$ denote the observed death toll and estimated death toll via the SIRDC model, respectively; The noise follows independently as a Gaussian distribution $\varepsilon _{i,j,t} \sim N(0, \sigma _{i,j,0}^2)$ with variance parameter $\sigma _{i,j,0}^2$. The latent temporal process $z_{i,j}(t)$ is modeled by a zero-mean GP, meaning that for time points $\{1, 2, \dots , T_{i,j}\}$, ${\mathbf {z}}_{i,j} = \left( z_{i,j}(1), \dots , z_{i,j}(T_{i,j})\right) ^T$ follows a multivariate normal distribution:

$$\begin{aligned} {\mathbf {z}}_{i,j} \sim \text{ MN }({\mathbf {0}}, \varvec{\Sigma }_{i,j}), \end{aligned}$$

where the (*l*, *m*) entry of $\varvec{\Sigma }_{i,j}$ is parameterized by a covariance function $\sigma ^2_{i,j} K_{i,j}(l, m)$ for $1\le l,m \le T_{i,j}$. Here $\sigma ^2_{i,j}$ is the variance parameter and $K_{i,j}(\cdot , \cdot )$ is a one-dimensional correlation function. We use the power exponential correlation function:

$$\begin{aligned} K_{i,j}(l, m) = \text{ exp }\left\{ -\left( \frac{\mid l - m \mid }{ b_{i,j}} \right) ^{a} \right\} , \end{aligned}$$

where *a* is the roughness parameter fixed to be 1.9 as in other studies^26,27^, to avoid possible singularity in inversion of the covariance matrix using the Gaussian correlation ($a=2$), and $b_{i,j}$ is a range parameter for each county estimated from the data. We define the nugget parameter $\eta _{i,j}=\sigma ^2_{i,j,0}/\sigma ^2_{i,j}$. The range parameter $b_{i,j}$, and the nugget parameter $\eta _{i,j}$ in Eq. (10) are estimated based on the marginal posterior mode estimation using the rgasp function in the package RobustGaSP available on CRAN^24^.

Denote ${\mathbf {D}}_{i,j}=(D_{i,j}(1),\ldots ,D_{i,j}(T_{i,j}))^T$and ${\mathbf {F}}_{i,j}=(F_{i,j}(1), \ldots ,F_{i,j}(T_{i,j}))^T$. After marginalizing out the variance parameter by the reference prior $p(\sigma ^2_{i,j})\propto 1/\sigma ^2_{i,j}$, for any $t^*$, the predictive distribution of $z_{i,j}({t}^*)$, conditional on the observations, range parameter $b_{i,j}$ and nugget parameter $\eta _{i,j}$, follows a non-central Student’s t-distribution with degrees of freedom $T_{i,j}$^24^

11 $$\begin{aligned} z_{i,j}\left( {t}^{*}\right) \mid {\mathbf {D}}_{i,j}, {\mathbf {F}}_{i,j}, b_{i,j}, \eta _{i,j} \sim {\mathscr{T}} \left( \hat{z}_{i,j}\left( {t}^{*}\right) , {\hat{\sigma }}^{2}_{i,j} {\tilde{K}}^{*}_{i,j}, T_{i,j}\right) , \end{aligned}$$

where

$$\begin{aligned} \begin{aligned} \hat{z}_{i,j}\left( {t}^{*}\right) =&F_{i,j}(t^*)+ {\mathbf {r}}^{T}_{i,j}\left( {t}^{*}\right) \tilde{{\mathbf {R}}}^{-1}_{i,j}({\mathbf {D}}_{i,j}- {\mathbf {F}}_{i,j}), \\ {\hat{\sigma }}^{2}_{i,j}=&\frac{ ({\mathbf {D}}_{i,j}- {\mathbf {F}}_{i,j})^T {\tilde{\mathbf {R}}}_{i,j}^{-1} ({\mathbf {D}}_{i,j}- \mathbf{F}_{i,j})}{T_{i,j}}, \\ {\tilde{K}}^{*}_{i,j}=&K_{i,j}\left( {t}^{*}, {t}^{*}\right) +\eta _{i,j}-{\mathbf {r}}^{T}_{i,j}\left( {t}^{*}\right) {\tilde{{\mathbf {R}}}}^{-1}_{i,j} {\mathbf {r}}_{i,j}\left( {t}^{*}\right) , \end{aligned} \end{aligned}$$

with ${\tilde{{\mathbf {R}}}}_{i,j}={\mathbf {R}}_{i,j}+\eta _{i,j}\mathbf{I}_{T_{i,j}}$, the (*l*, *m*)th term of ${\mathbf {R}}_{i,j}$ being $K_{i,j}(l,m)$ for $1\le l,m\le T_{i,j}$, and ${\mathbf {r}}_{i,j}(t^*) = (K_{i,j}({t}^*, 1), \dots , K_{i,j}({t}^*, T_{i,j}))^{T}$, by plugging in the estimated range parameter $b_{i,j}$ and nugget $\eta _{i,j}$. The predictive mean ${\hat{z}}_{i,j}(t^*)$ for forecasting the death toll of the *j*th county in the *i*th state at a future day $t^*$ and the predictive interval can be computed based on the Student’s *t* distribution. An overview of the forecast algorithm and the numerical comparison of different approaches in forecast is given in the Supplementary Information.

## Supplementary Information

Supplementary Information.

Supplementary Video 1.

Supplementary Video 2

Supplementary Video 3.

## Data Availability

The datasets analysed in the current study are available in the CSSEGISandData repository, https://github.com/CSSEGISandData/COVID-19 and COVID-19 data tracking project, https://covidtracking.com/. The US maps are graphed based on publicly available R package urbnmapr. The code used in this paper is publicly available: https://github.com/HanmoLi/Robust-estimation-of-SARS-CoV-2-epidemic-in-US-counties/.
