# Supplementary material for: Robust estimation of SARS-CoV-2 epidemic in US counties
Source: Sci Rep. 2021 Jun 4;11:11841. doi: 10.1038/s41598-021-90195-6 (PMC8178310; doi:10.1038/s41598-021-90195-6)
Supplement: Supplementary file 1 — Supplementary Information. [file 41598_2021_90195_MOESM1_ESM.pdf]

Robust estimation of SARS-CoV-2 epidemic in US counties

Hanmo Li[1,+] and Mengyang Gu[1,*,+]

[1]Department of Statistics and Applied Probability, University of California, Santa Barbara, California, USA, 93106
[*]mengyang@pstat.ucsb.edu
[+]the authors contributed equally to this work

Supplementary materials

The supplementary materials contain three parts. In the first part, We discuss the details of model parameter specification and conduct a sensitivity analysis. The forecast algorithm and numerical comparison of different approaches are introduced in the second part. The third part contains three videos for the county-level estimation of the daily PoC SARS-CoV-2 of a susceptible individual, the effective reproduction numbers, and the number of active infectious individuals from 21 March 2020 to 20 September 2020.

S1 Model parameter specification and sensitivity analysis

We discuss the choice of the model parameters and their sensitivity analysis. The following parameters of the SIRDC model were specified based on previous studies.

- The death rate or the infection fatality ratio (δ) that measures the proportion of death among all infected individuals. We assume $\delta = 0.66\%$ following[1].
- The inverse of the number of days an infectious individual can transmit the COVID-19 (γ). The average time of a COVID-19 patient to transmit disease is assumed to be 5 days in[2], indicating that $\gamma = 0.2$. Another evidence comes from the study of the incubation period. The latent period (exposed but not contagious) for COVID-19 is found to be 3.69 days on average[3] and the mean incubation period (time from infection to onset of symptoms) is 5.2 days[4], meaning that the infectious period is around 1.5 days before the onset of symptom. The diagnostic test could take less than one day to up to a week. We thus assume 3.5 days to get the result of a diagnostic test on average. The total infectious period is around 5 days.
- The inverse of the number of dates for resolving case to get resolved (θ). According to the CDC report[5], for mild and moderate symptom, the replication-competent virus has not been recovered after 10 days following symptom onset, indicating the individuals remains infectious no longer than 10 days after symptom onset. The infectious period could be as long as 20 days for patients with more severe illness from COVID-19 infection. Since a majority of the COVID-19 infections are mild to moderate, we assume the infectious period to be 13.5 days, and after reducing 3.5 days from onset of the symptom to become resolving (after quarantine or hospitalization), it takes around 10 days for a resolving case to resolved on average.

We conduct a sensitivity analysis to examine the change of the estimation in 4 different configurations.

- (**Configuration 1**) $(\gamma, \theta, \delta) = (0.2, 0.1, 0.0066)$, the default parameter set.
- (**Configuration 2**) $(\gamma, \theta, \delta) = (0.14, 0.1, 0.0066)$. The average length of infectious period changes from 5 days to $\frac{1}{0.14} \approx 7$ days, whereas other parameters are held unchanged.
- (**Configuration 3**) $(\gamma, \theta, \delta) = (0.2, 0.067, 0.0066)$. The average length of resolving period changes from 10 days to $\frac{1}{0.067} = 15$ days, whereas other parameters are held unchanged.
- (**Configuration 4**) $(\gamma, \theta, \delta) = (0.2, 0.1, 0.0075)$. The infection fatality ratio changes from 0.66% to 0.75%, whereas other parameters are held unchanged.

After specifying the parameters (γ, θ, δ), the transmission rate $\beta(t)$ can be obtained from algorithm 1. Figure S1 gives result of the sensitivity analysis. First, we found the estimated death toll for 4 scenarios is almost the same (part d in Figure

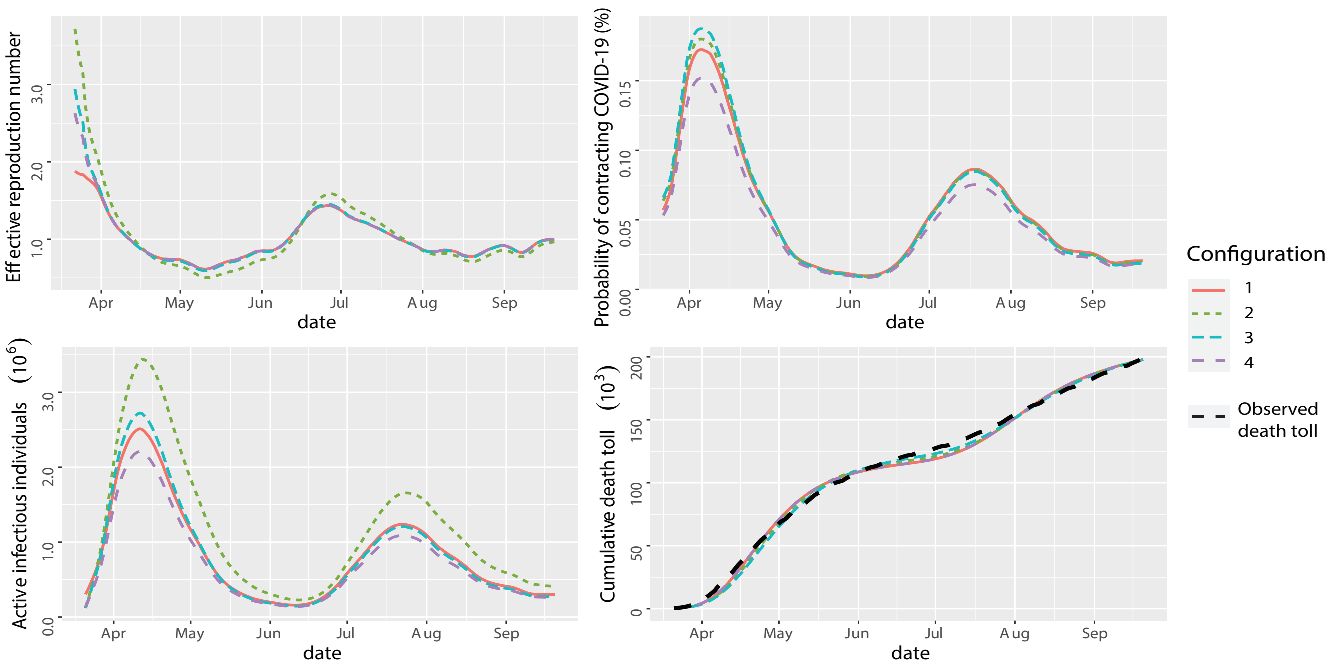

Figure S1. sensitivity analysis for 4 configurations of the SIRDC model parameters. Part a-d shows the estimated effective reproduction number, PoC SARS-CoV-2, the number of active infectious individuals, and cumulative death toll, respectively.

S1). Extending the infectious period from 5 to 7 days (Configuration 2) increases the number of active infectious individuals and effective reproduction number shown in part a and part c in Figure S1, respectively. Consequently, the peak of average daily PoC SARS-CoV-2 slightly increases in the first wave, whereas the scale of increase is smaller than the change in the effective reproduction number and active infectious individuals. The average daily PoC SARS-CoV-2 has almost no change in other periods, indicating that the length of the average infectious period has almost no influence of our estimation on PoC SARS-CoV-2 for most of the days.

Second, when the average length of the resolving period changes from 10 to 15 days, the peak of PoC SARS-CoV-2, effective reproduction number, and the number of active infectious individuals increases in the first wave, whereas these quantities remain largely unchanged for the rest of the days (part a-c in Figure S1). The result indicates that the average length of the resolving period also barely affects the estimated characteristics of COVID-19 progression for most of the days.

When the death rate increases from 0.66% to 0.75%, the effective reproduction number seems to have almost no change (part a in Figure S1), whereas the PoC SARS-CoV-2 and the number of active infectious individuals (figure S1 part b-c) both reduce. This is because when the death rates increase, the estimated number of individuals infected decreases, as the death toll is observed (and thus fixed). The death rate is a key parameter to calibrate, and studies of the prevalence of SARS-CoV-2 antibodies based on serology tests[6] can be used to estimate the death rate in each state.

In conclusion, parameter values of the average length of the infectious period and the average length of the resolving period barely change the COVID-19 progression characteristics for most of the days, including the fitted death toll. On the other hand, we found that the number of active infectious individuals and the daily PoC SARS-CoV-2 depend critically on the death rate parameter. Further studies of prevalence would be useful for estimating the death rate **parameter** in different regions.

S2 Algorithm of Forecast and numerical comparison

An overview of our algorithm for forecast and uncertainty assessment is given in algorithm S1, where inputs are the county-level observed cumulative number of confirmed cases $\mathbf{c}^o_{i,j} = (c^o_{i,j}(1), \ldots, c^o_{i,j}(T_{i,j}))^T$, the county-level observed cumulative death toll $\mathbf{D}_{i,j}$, the state-level test positive rate $\mathbf{p}_i = (p_i(1), \ldots, p_i(T_i))^T$, state-level confirmed cases $\mathbf{c}^o_i = (c^o_i(1), \ldots, c^o_i(T_i))^T$ and state-level death toll $\mathbf{D}_i = (D_i(1), \ldots, D_i(T_i))^T$.

To evaluate the performance of different approaches, we implement 7-day and 21-day forecasts on 2,277 US counties with a training period from 21 March 2020 to 20 September 2020, and with the forecast period starting from 21 September 2020. To compare the prediction performance of different methods, we computed the rooted mean square error (RMSE), the proportion of the observations covered in the 95% predictive interval ($P_{CI}(95\%)$) and length of the 95% confidence interval ($L_{CI}(95\%)$),

Data: $\mathbf{c}^o_{i,j}$, $\mathbf{D}_{i,j}$, $\mathbf{p}_i$, $\mathbf{c}^o_i$, and $\mathbf{D}_i$.

Result: Estimates of county-level epidemiological compartments $\hat{\boldsymbol{\beta}}_{i,j}$, $\hat{\mathbf{S}}_{i,j}$, $\hat{\mathbf{I}}_{i,j}$, $\hat{\mathbf{R}}_{i,j}$, $\hat{\mathbf{C}}_{i,j}$, forecast $\hat{\mathbf{D}}^*_{i,j}$, where $\hat{\mathbf{D}}^*_{i,j} := \left(\hat{D}_{i,j}(T_{i,j}+1),\ldots,\hat{D}_{i,j}(T_{i,j}+T^*)\right)^T$, and the uncertainty assessment of the compartments.

Step 1 Conduct a three-parameter constrained optimization treating state-level power parameter α_i unknown to minimize the loss function in equation (9) using $\mathbf{p}_i$, $\mathbf{c}^o_i$ and $\mathbf{D}_i$.

Step 2 For each county, set initial values $I_{i,j}(1) = R_{i,j}(1) = 1,000$, $C_{i,j}(1) = 0$ and $D_{i,j}(1)$ to be the observed death toll on day 1. Find the optimized values of $I_{i,j}(1)$ and $R_{i,j}(1)$ to minimize equation (9).

Step 3 Simulate $S = 500$ samples of the observed confirmed cases sampled from the predictive distribution of a GP model of the observed confirmed cases. For each sample, obtain the other compartments and time-dependent transmission rate by equation (1)-(5) and algorithm 1 using the estimate of the initial values.

Step 4 Extrapolate the time-dependent transmission rate parameters from a GP model for each sample and obtain $S = 500$ samples of the output death toll of the SIRDC at the forecast period.

Step 5 Sample the residuals from the predictive distribution in Equation (11) in the main manuscript at the forecast period and obtain $S = 500$ samples of the ensemble forecast for the death toll. Compute the mean for forecast and 95% predictive interval to quantify uncertainty of forecast.

Algorithm S1: Ensemble forecast and uncertainty assessment.

defined as follows:

$$\text{RMSE} = \sqrt{\frac{\sum_{i=1}^{k}\sum_{j=1}^{n_i}\sum_{s\in\mathbf{t}^*}(\hat{D}_{i,j}(s) - D_{i,j}(s))^2}{\sum_{i=1}^{k} n_i T^*}}$$

$$P_{CI}(95\%) = \frac{1}{\sum_{i=1}^{k} n_i T^*}\sum_{i=1}^{k}\sum_{j=1}^{n_i}\sum_{s\in\mathbf{t}^*}\mathbb{1}_{\{D_{i,j}(s)\in CI_{i,j,s}(95\%)\}}$$

$$L_{CI}(95\%) = \frac{1}{\sum_{i=1}^{k} n_i T^*}\sum_{i=1}^{k}\sum_{j=1}^{n_i}\sum_{s\in\mathbf{t}^*}\text{length}\{CI_{i,j,s}(95\%)\}$$

where $\mathbf{t}^* := (T_{i,j}+1,\ldots,T_{i,j}+T^*)$, $T^* = 7$ and $T^* = 21$ for the 7-day forecast and 21-day forecast, respectively. A model with small RMSE, $P_{CI}(95\%)$ close to the nominal 95% and small $L_{CI}(95\%)$ is precise for forecast and uncertainty assessment.

A comparison between our approach and the other three approaches is recorded in Table S1. Our approach (denoted in SIRDC+GP) has the lowest RMSE among 4 methods considered herein. Approximately 95% of the held-out death toll are covered by the 95% predictive interval by our approach, indicating our uncertainty assessment is accurate. Although other approaches produce a shorter length of the predictive interval, the number of held-out observations in the 95% predictive interval is smaller than ours. Therefore, combining the SIRDC model and GP for modeling the residuals may improve the predictive accuracy for forecasting COVID-19 associated death toll at US counties, compared to the one using the SIRDC model or the GP model alone.

Prediction period	Method	RMSE	$P_{CI}(95\%)$	$L_{CI}(95\%)$
7 days	SIRDC+GP	**3.04**	95.06%	23.05
	SIRDC	4.12	/	/
	GP without linear trend	3.18	91.29%	**4.82**
	GP with linear trend	4.36	88.28%	5.51
21 days	SIRDC+GP	**6.81**	93.46%	28.37
	SIRDC	7.79	/	/
	GP without linear trend	7.20	92.14%	**11.74**
	GP with linear trend	11.93	76.94%	10.18

Table S1. 7-day and 21-day forecast in 2,277 US counties with training period from 21 March 2020 to 20 September 2020 and with prediction period starting from 21 September 2020. Four methods are compared. Our proposed approach that combines the SIRDC model and a zero-mean GP to model the residuals is denoted as SIRDC+GP. Second, the death forecast by SIRDC model is denoted as SIRDC, which contains Steps 1 and 2 in the algorithm S1 and provides point projection of the death toll. Third, a GP with a constant mean function is denoted as GP without linear trend, which equivalently replaces the SIRDC model of a constant mean parameter estimated by the data for each county. The fourth model is the same as the third method, except that the mean of GP contains a constant mean and a linear trend of time with two linear coefficient parameters estimated from the data (denoted as GP with linear trend). The method of the best performance by each criterion is highlighted.

References

1. Verity, R. *et al.* Estimates of the severity of coronavirus disease 2019: a model-based analysis. *The Lancet infectious diseases* **20**, 669–677 (2020). https://doi.org/10.1016/S1473-3099(20)30243-7.
2. Davies, N. G. *et al.* Effects of non-pharmaceutical interventions on COVID-19 cases, deaths, and demand for hospital services in the UK: a modelling study. *The Lancet Public Heal.* **5**, e375 – e385 (2020). https://doi.org/10.1016/S2468-2667(20)30133-X.
3. Li, R. *et al.* Substantial undocumented infection facilitates the rapid dissemination of novel coronavirus (SARS-CoV-2). *Science* **368**, 489–493 (2020). https://science.sciencemag.org/content/368/6490/489.
4. Li, Q. *et al.* Early transmission dynamics in Wuhan, China, of novel coronavirus–infected pneumonia. *New Engl. J. Medicine* (2020). https://doi.org/10.1056/NEJMoa2001316.
5. for Disease Control, C., Prevention *et al.* Duration of Isolation and Precautions for Adults with COVID-19. *Atlanta, GA: CDC* (2020).
6. Anand, S. *et al.* Prevalence of SARS-CoV-2 antibodies in a large nationwide sample of patients on dialysis in the USA: a cross-sectional study. *The Lancet* **396**, 1335–1344 (2020). https://doi.org/10.1016/S0140-6736(20)32009-2.
